# Patients’ satisfaction with healthcare services in Al-Baha, Saudi Arabia: a cross-sectional study

**DOI:** 10.25122/jml-2024-0391

**Published:** 2025-02

**Authors:** Ramy Hassan Agwa, Turki Alkully, Warda Othman, Sahar Abdulrahman Alghamdi, Haya Abdulaziz Alzahrani, Nada Saleh Algamdi, Yahya Saeed Al Zahrani, Abdulrhman Saleh Alzhrani

**Affiliations:** 1 Internal Medicine Department, Faculty of Medicine, Al-Baha University, Al-Baha, Saudi Arabia; 2 Internal Medicine Department, Faculty of Medicine, Mansoura University, Mansoura, Egypt; 3 Department of Hepatology and Gastroenterology, National Liver Institute, Menoufia University, Menoufia, Egypt; 4 Faculty of Medicine, Al-Baha University, Al-Baha, Saudi Arabia

**Keywords:** satisfaction, Al-Baha, healthcare, quality, Saudi Arabia

## Abstract

Patient satisfaction is crucial in assessing healthcare quality, encompassing factors such as continuity of care, waiting times, and physician-patient communication. This study evaluated patient satisfaction with healthcare services and determined the main reasons for low self-reported quality. A cross-sectional study was conducted in the Al-Baha region, including Saudi adults aged 18 to 60 who visited public or private health centers. Data were collected using a modified patient satisfaction questionnaire. The Chi-square test and logistic regression were utilized. Out of 388 participants, 55.2% were women. Most were highly educated and resided in Al-Baha. Long waiting times (38.4%) and appointment difficulties (25.8%) were the most common drawbacks. Men and Al-Baha residents had higher satisfaction scores. Higher income was linked to better accessibility and convenience scores. Satisfaction was higher among patients at private hospitals. The study provides insights into patient satisfaction in Al-Baha, highlighting the need to reduce waiting times and improve appointment systems to enhance healthcare quality.

## INTRODUCTION

Since healthcare systems are constantly evolving, it is important to measure patient satisfaction alongside healthcare outcomes [1]. Patient satisfaction describes how individuals perceive the services that the healthcare system provides to meet their needs [2]. Patients' expectations of their healthcare providers and their perceived requirements may not always coincide. Therefore, patients' experiences with their healthcare services are key variables influencing healthcare service utilization [3]. Several modifiable factors affect patient satisfaction, including continuity of care, reduced waiting times, and promoting physician-patient communication [4]. Multiple studies, including one in the Indian state of West Bengal, showed that the total satisfaction rate was 73.1%, with a mean value of 3.655. The domains with the highest and lowest levels of satisfaction were general satisfaction and time spent with doctors [5]. Similarly, a study in Malaysia assessed factors influencing patient satisfaction and trust among individuals who visited general practice clinics. The findings revealed that while external design was not linked to patient satisfaction or trust, ambiance, service delivery, interior decor, and cleanliness significantly impacted the patients' sense of trust and satisfaction [6]. A comparable study conducted in 2023 among 384 patients at two tertiary care facilities in Riyadh found that 73.77% of participants were generally satisfied with all 18 measured aspects [7]. The present study aimed to track patient satisfaction levels in relation to healthcare system quality using a satisfaction index. Additionally, it evaluated the influence of socioeconomic and medical service components [8]. Research on patient satisfaction in the Kingdom of Saudi Arabia has shown varying levels among patients [9]. However, no studies have specifically evaluated patient satisfaction in the Al-Baha region. Therefore, the primary goal of this study was to evaluate the degree of patient satisfaction with medical care in the Al-Baha area and determine key factors contributing to lower self-reported healthcare quality.

## MATERIAL AND METHODS

### Study design

This descriptive cross-sectional study was conducted in the Al-Baha region from November 2023 to March 2024 to evaluate the satisfaction levels of Saudi residents with various healthcare services and to explore the main reasons associated with challenges in achieving optimal quality.

### Study population

The study population consisted of all Saudi male and female adult patients aged 18 to under 60 who were Arabic speakers and had visited any public or private health center at least once within the past year. Individuals below 18 or above 60 years, medical field workers (including medical administration staff, physicians, nurses, and laboratory specialists), non-Arabic speakers, and non-Saudis were excluded.

### Sample size

The required sample size was estimated using the following formula:

*n* = P (1-P) * Zα 2 / d^2^

Where:

*n* = calculated sample size

Z = Z-value for a 95% confidence level = 1.96

P = Estimated knowledge

Q: (1 – 0.50) = 50%, i.e., 0.50

D: The maximum acceptable error = 0.05

The minimum required sample size was

*n* = (1.96)^2^ × 0.50 × 0.50/(0.05)^2^ = 384 [7].

### Data collection tool

Data were collected using a modified version of the Patient Satisfaction Questionnaire-18 (PSQ-18) to assess patient satisfaction [10,11]. The questionnaire consisted of three sections. The first section included demographic information such as age, gender, residence, occupation, income, type of healthcare institution, point of access (ER department or outpatient clinics), and the reason for seeking medical attention. The second section comprised 18 closed-ended questions that evaluated patients' satisfaction with medical services in seven primary areas: communication, interpersonal manner, technical quality, general satisfaction, financial aspects, time spent with the doctor, accessibility, and convenience. The sum of all subscale scores was calculated. For each question, participants selected one of five responses: strongly agree, agree, uncertain, disagree, and strongly disagree. Each response was assigned a score from one to five, with five denoting the highest level of satisfaction: five points for strongly agree, four for agree, three for uncertain, two for disagree, and one for strongly disagree. This scoring system is the opposite of the original PSQ-18, where lower scores on each subscale indicate better healthcare performance and, ultimately, greater patient satisfaction. These alterations were made for easier comprehension and score calculations, especially among illiterate individuals. Additionally, the authors added a final section to include common healthcare disadvantages. The questionnaire was translated into Arabic by sixth-year medical students, and the translation was verified by two individuals with bachelor's degrees in English Language and Literature. It was then formulated into an online survey using Google Forms. The questionnaire was disseminated through several social media platforms to reach target populations. A pilot study was conducted with twenty individuals to assess the validity and clarity of the questions. No further modifications were made based on the pilot study data, and the participants confirmed the clarity of the questionnaire by providing short answers to an open-ended question regarding their comprehension of questions.

### Statical analysis

All collected data were entered into the Statistical Package for the Social Sciences (SPSS) version 26. Quantitative measures, comprising the mean and standard deviation (SD), were used to categorize participants. The satisfaction score was separated into two categories: dissatisfaction (equal to or below the mean) and satisfaction (above the mean). The relationship between satisfaction across various categories and socioeconomic backgrounds was examined using the Chi-square test. The Mann-Whitney test was used to assess differences between groups with higher and lower satisfaction, as the normality test indicated that the scores were not normally distributed. To identify predictors influencing the relationship between various factors and patients' satisfaction levels, logistic regression analysis was utilized. A *P* value of <0.05 was considered statistically significant for all analyses.

## RESULTS

### Sociodemographic features and healthcare experience of the participants

A total of 388 participants were recruited for the study. Of these, 55.2% were women, and the most common age group was 21–30 years (43%), with a mean age of 31 ± 11 years. The majority of participants (74%) had a high education level, and 52.6% resided in Al-Baha. Most participants were not healthcare workers (69.3%, Table 1).

**Table 1:** Sociodemographic characteristics of the study participants (*n* = 388)

Sociodemographic characteristics	*n* (%)	Percentage
**Age group**		
Mean ± SD (years)	31.1 ± 11.4	
15-20	58	15%
21-30	167	43%
31-40	80	20.6%
High than 40	83	21.4%
**Gender**		
Male	174	44.8%
Female	214	55.2%
**Residence**		
Al Baha	204	52.6%
Other	184	47.4%
**Healthcare worker**		
Yes	13	3.4%
No	269	69.3%
Medical student	106	27.3%
**Educational level**		
Secondary	101	26%
Higher	287	74%
**Income**		
Less than 5000	201	51.8%
5000 or higher	187	48.2%

Among the participants, 77.1% (*n* = 299) were first-time visitors, while the remaining attended follow-up visits. The majority (66%, *n* = 256) received care at government hospitals, followed by 23.5% (*n* = 91) who visited healthcare centers. Regarding perceived disadvantages of healthcare services, the most commonly reported issue was long waiting times (38.4%, *n* = 149), followed by difficulty in appointment scheduling (25.8%, *n* = 100; Table 2).

**Table 2:** Healthcare experience among study participants (*n* = 388)

Variable	*n*	Percentage
**Type of visit**		
First visit	299	77.1%
Follow up	89	22.9%
**Type of hospital**		
Health care center	91	23.5%
Governmental hospital	256	66%
Private hospital	41	10.5%
**Health care disadvantages**		
High cost	25	6.4%
Health care provider attitude	61	15.7%
Difficulty in appointment	100	25.8%
Low service	53	13.7%
Long waiting time	149	38.4%

### Patient satisfaction subscales and comparison between groups

Patient satisfaction was assessed across seven subscales, each containing 2 to 4 items: general satisfaction, technical quality, interpersonal aspect, communication, financial aspect, time spent with doctor, and accessibility and convenience.

Participants were categorized based on their mean total satisfaction score, with those scoring above the mean classified as high satisfaction and those scoring below the mean as low satisfaction. The mean satisfaction score for the general satisfaction subscale was 9.5 ± 0.7 in the high satisfaction group and 6.8 ± 1.8 in the low satisfaction group. Similarly, the technical quality subscale had mean scores of 19 ± 1.4 for the high satisfaction group and 13 ± 3.3 for the low satisfaction group. Detailed scores for other subscales are presented in Table 3. After applying the Mann–Whitney test, the results showed a statistically significant difference between both groups regarding all subscales (*P* < 0.05).

**Table 3:** Patient satisfaction subscales across study groups (*n* = 388)

Domain	Number of items	Mean ± SD		*P* value
		High satisfaction	Low satisfaction	
**General satisfaction (out of 10)**	2	9.5 ± 0.7	6.8 ± 1.8	0.001
**Technical quality (out of 20)**	4	19 ± 1.4	13 ± 3.3	0.001
**Interpersonal aspect (out of 10)**	2	9.5 ± 0.9	7.2 ± 1.6	0.001
**Communication (out of 10)**	2	9.5 ± 0.7	7 ± 1.6	0.001
**Financial aspect (out of 10)**	2	9.5 ± 0.9	7.2 ± 1.9	0.001
**Time spent with doctor (out of 10)**	2	9.3 ± 1.1	6.2 ± 1.7	0.001
**Accessibility and convenience**	4	18.8 ± 1.8	13.1 ± 3.3	0.001

### Comparison of sociodemographic characteristics with patient satisfaction subscales

A Chi-square test was used to examine the relationship between satisfaction subscales and sociodemographic factors (Table 4). Male participants had significantly higher satisfaction scores across all subscales compared to females (*P* < 0.05). Also, residents of Al Baha reported higher satisfaction scores than residents of other towns in all subscales (*P* < 0.05). Furthermore, participants with an income above 5000 SAR scored significantly higher (*P* < 0.05) in the accessibility and convenience subscale. All details are shown in Table 4.

**Table 4:** Comparison of patient satisfaction subscales with sociodemographic characteristics (*n* = 388)

Variable	General satisfaction (Mean ±SD)	Technical quality (Mean ±SD)	Interpersonal aspect (Mean ±SD)	Communication (Mean ±SD)	Financial aspect (Mean ±SD)	Time spent with doctors (Mean ±SD)	Accessibility and convenience (Mean ±SD)	Overall (Mean ±SD)
**Age group**								
15-20	8.4 ± 2	16.8± 3.9	8.4 ± 1.8	8.4± 2	8.7 ± 1.8	8 ± 2.1	16.8 ± 4.3*	75.6± 16.2
21-30	8.3 ± 2	16 ± 4.1	8.4 ± 1.7	8.3± 1.8	8.6 ± 1.8	7.8 ± 2.2	16.2± 4.1	73.7± 15.1
31-40	8.3 ± 1.5	16.6 ± 3	8.4 ± 1.4	8.3 ± 1.4	8.1 ± 1.6	8.1± 1.7	16.4± 3	74.3± 11.5
High than 40	8.2 ± 1.8	16.5± 3.7	8.4 ± 1.7	8.4 ± 1.7	8.3 ± 2	7.8 ± 2.1	15.8 ± 3.9	73.5± 15.5
**Gender**								
Female	8± 2*	15.8± 4*	8.2± 1.8*	8.1± 1.9*	8.2 ± 2*	7.5± 2.2*	15.5± 4.1*	71.2 ± 15.3*
Male	8.6 ± 1.6	17.1 ± 3.4	8.7 ± 1.4	8.6 ± 1.5	8.8 ± 1.6	8.4 ± 1.8	17.2 ± 3.2	77.5± 13
**Residence**								
Al Baha	8.5 ± 1.7*	17 ± 3.6*	8.7 ± 1.5*	8.5 ± 1.7*	8.7 ± 1.5*	8.3 ± 2 *	17 ± 3.6*	76.7 ± 13.7 *
Others	8 ± 2	15.7 ± 4	8.2 ± 1.8	8.1 ± 1.8	8.2 ± 2	7.4 ± 2	15.5 ± 4	71.1 ± 15.1
**Educational level**								
Secondary	8.3 ± 1.7	16.6± 3.3	8.3 ± 1.5	8.4 ± 1.6	8.4 ± 1.7	7.8± 2	16.4± 3.5	74.3 ± 13
Higher	8.3 ± 1.9	16.3± 4	8.4 ± 1.7	7.3 ± 1.8	8.5 ± 1.9	8 ± 2.1	16.2± 4	74 ± 15
**Income**								
Less than 5000	8.3± 2	16.3 ± 3.8	8.4 ± 1.6	8.3 ± 1.8	8.5 ± 1.7	7.8± 2	16.2± 4*	74 ± 14.3
5000 or higher	8.3± 1.7	16.4 ± 3.7	8.4 ± 1.7	8.4 ± 1.7	8.4± 1.9	8 ± 2	16.3 ± 3.7	74.2 ± 15
**Type of visit**								
First visit	8.3 ± 1.8	16.3 ± 3.7	8.4 ± 1.6	8.3 ± 1.7*	8.5 ± 1.7*	7.9 ± 2	16.4 ± 3.6	74.1 ± 13.9
Follow up	8.2 ± 2	16.6 ± 4	8.5 ± 2	8.5 ± 2	8.3 ± 2	8 ± 2.3	16 ± 4.5	73.9 ± 17
**Type of hospital**								
Health care center	8.1 ± 1.8	15.7 ± 4	8 ± 1.7	8 ± 1.9	8.1 ± 2	7.6 ± 2	15.8 ± 3.9	71.4 ± 15.4
Governmental hospital	8.3± 2	16.5 ± 3.8	8.5 ± 1.7	8.5 ± 1.8	8.6 ± 1.8	8 ± 2	16.2 ± 3.9	74.6 ± 14.8
Private hospital	8.4 ± 1.6	16.8 ± 2.8	8.5 ± 1.4	8.5 ± 1.4	8.6 ± 1.8	8.4± 1.9	17.3± 3	76.6 ± 11.4

* *P* value is significant less than 0.05

### Predictors of patient satisfaction

Logistic regression analysis was used to identify the predictors that affect the relation between different sociodemographic features and satisfaction. Male participants were 3.1 times more likely to report satisfaction with medical services than females (*P* = 0.001). Al-Baha residents were 2.8 times more likely than other residents to report satisfaction with the medical service (*P* = 0.001). Regarding the type of hospital, private hospital patients were 2.7 times more likely to report satisfaction than those visiting healthcare centers (*P* = 0.012). Government hospital patients were 1.9 times more likely to report satisfaction than healthcare center patients (*P* = 0.009, Table 5).

**Table 5:** Logistic regression analysis

Variables	Frequency (%)		Odds ratio	Confidence interval	*P* value
	Satisfied *n* = 213	Non-satisfied *n* = 175			
**Age group**					
15-20	38(17.9%)	20(11.5%)	1.7	0.8-3.4	0.1
21-30	88(41.3%)	79(45.1%)	1	0.6-1.7	0.9
31-40	42(19.7%)	38(21.7%)	1	0.5-1.9	1
High than 40	45(21.1%)	38(21.7%)	Reference	-	-
**Gender**					
Female	91(42.7%)	123(70.3%)	Reference		
Male	122(57.3%)	52(29.7%)	3.1	0.2-4.8	0.001
**Residence**					
Al Baha	137(64.3%)	67(38.3%)	2.8	1.8-4.3	0.001
Others	76(35.7%)	108(61.7%)	Reference	-	-
**Health care worker**					
Yes	7(3.3%)	6(3.4%)	1.3	0.4-4.3	0.6
No	157(73.7%)	112(64%)	1.6	1-2.6	0.03
Medical student	49(23%)	57(32.6%)	Reference	-	-
**Educational level**					
Secondary	57(26.8%)	44(25.1%)	1.1	0.7-1.7	0.7
Higher	156(73.2%)	131(74.9%)	Reference	-	-
**Income**					
Less than 5000	108(50.7%)	93(53.1%)	Reference		
5000 or higher	105(49.3%)	82(46.9%)	1	0.6-1.5	0.9
**Type of visit**					
First visit	158(74.2%)	141(80.6%)	Reference		
Follow up	55(25.8%)	34(19.4%)	1.2	0.7-2.1	0.4
**Type of hospital**					
Health care center	38(17.8%)	53(30.3%)	Reference		
Governmental hospital	148(69.5%)	108(61.7%)	1.9	1.2-3	0.009
Private hospital	41(12.7%)	14(8%)	2.7	1.2-5.8	0.012

## DISCUSSION

The primary objective of this study was to evaluate the general population’s perception of healthcare services and the level of patient satisfaction in the Al-Baha region of Saudi Arabia. Patient satisfaction was assessed using the PSQ-18 scale, covering multiple subscales. A greater proportion of female participants engaged with the online survey, which may indicate that more women are aware of the significance of collaborating in various medical studies to create a local database and address the pitfalls of healthcare, similar to the findings of Chakraborty *et al*. [5]. However, these findings contrast with a large retrospective cross-sectional study conducted in Saudi Arabia, which examined satisfaction with the service quality of primary healthcare centers and involved fewer women [14]. These differences may be attributed to variations in sociodemographic data and different modalities of questionnaire distribution.

Socioeconomic factors play a crucial role in shaping healthcare access and patient satisfaction, influencing the overall quality of care received [15]. These factors include income, education, geographic location, type of visit, and healthcare facility. In our study, male residents of Al-Baha city scored significantly higher on all PSQ-18 subscales than females and those from other towns in the Al-Baha region, such as Almikhawah and Alaqiq, indicating that urbanization is associated with better quality services based on PSQ-18 findings. Our results extend the findings of a systematic review that identified individual characteristics as possible determinants of patient satisfaction, as the satisfied groups were predominantly young men [16,17].

These findings contradict a cross-sectional study by Almass *et al*. in 2022, which involved 2,997 patients admitted to emergency departments in multiple hospitals across various regions of the Kingdom of Saudi Arabia, highlighting that men had worse satisfaction scores than women [4]. The association between patient satisfaction and urban areas has been illustrated in a study exploring the gap between rural and urban healthcare services in Egypt [18]. This study comprised a total of 693 patients (326 from urban areas and 367 from rural areas) and showed that a greater focus should be placed on certain aspects of healthcare delivery in rural governorates, such as nurse clinical and non-clinical training, infrastructure enhancements, and upgrades to the registration system.

This outcome may be attributed to Al-Baha city’s status as the regional capital, advanced medical infrastructure, and reputation as a popular tourist destination in Saudi Arabia. These factors, combined with diverse social and professional opportunities, contribute to the recruitment and retention of highly qualified physicians and their families. Additionally, the presence of Al-Baha University, which houses the Faculties of Medicine, Pharmacy, and Applied Medical Sciences, plays a pivotal role in strengthening the local healthcare workforce. Integrating university graduates and faculty members into the medical sector through residency and contractual agreements with healthcare institutions further enhances the availability of specialized medical services within the region.

Surprisingly, there was no association between the type of healthcare sector, whether public or private, and satisfaction across all subscales, including communication, interpersonal manner, technical quality, general satisfaction, financial aspects, time spent with doctors, accessibility, and convenience. However, patients with an income of 5000 SR or more scored significantly (*P* < 0.05) higher on the accessibility and convenience subscale than those with lower incomes, supporting the impact of financial income. This aligns with the findings of Xesfingi *et al*. [8], which indicated a strong positive correlation between patient satisfaction and income. Participants with higher incomes had better scores, specifically on the accessibility and convenience subscales, likely due to their ability to access specialized medical care and superior healthcare services, resulting in more effective and individualized treatment across the Al-Baha region. Similarly, a study analyzing data from a developed country found a negative correlation between long-term unemployment and patient satisfaction for both men and women [19]. We observed a weak correlation between the type of hospital, financial aspects, and time spent with doctors. This correlation was more thoroughly investigated by Mutiarasari *et al*. [20] with a larger sample size of 1,070 participants, comparing satisfaction levels in public and private hospitals. Their study revealed significant differences in satisfaction degrees related to requirements, procedures, service time, fees, product specifications, competency, attitudes, service notifications, and handling complaints, recommendations, and feedback (*P* < 0.05). This may indicate that patients who attended private hospitals were more satisfied with the medical services provided, received more attention and time from their healthcare providers, and recognized the importance of active listening from their treating doctors, contributing to better clinical outcomes and higher patient satisfaction.

One of the key strengths of this study is that it is the first to assess patient satisfaction in the Al-Baha region while considering various demographic and socioeconomic factors such as age, gender, education level, geographical location, type of visit, and hospital type. The levels of satisfaction were calculated based on well-established objective data collection tools used in similar studies. Precise inclusion and exclusion criteria were applied, and reliable ethical approval was obtained from senior researchers at the Al-Baha Faculty of Medicine.

The main limitation of this study may include recall bias, as no timeframe was utilized to evaluate patients' responses based on the time they visited the healthcare institutions. A cross-sectional study is not the best observational design to establish a relationship between certain variables. Healthcare providers were excluded, leading to a poor understanding of the acceptable level of care that must be provided according to national and international guidelines. Future research can compare the satisfaction levels of Saudi and non-Saudi patients and correlate healthcare workers' views with satisfaction levels. While our findings depend on a single province of Saudi Arabia, they may not represent the whole Saudi population. Therefore, the necessity of more studies and larger sample size would offer more representative findings of Al-Baha region citizens and the entire Saudi community.

## CONCLUSION

This study provides a foundational understanding of patient satisfaction and its implications in the Al-Baha region, Saudi Arabia. We identified key factors influencing patient satisfaction, including sociodemographic characteristics, healthcare facility type, and financial aspects, and examined their impact on patients’ perceptions of care quality. We consider reducing waiting times and facilitating outpatient clinic appointments as fundamental aspects to enhance care in the region.

## Personal thanks

All authors express their sincere gratitude to the citizens of the Al-Baha region for their collaboration.
